# Impact of MUC1 Mucin Downregulation in the Phenotypic Characteristics of MKN45 Gastric Carcinoma Cell Line

**DOI:** 10.1371/journal.pone.0026970

**Published:** 2011-11-02

**Authors:** Natália R. Costa, Paula Paulo, Thomas Caffrey, Michael A. Hollingsworth, Filipe Santos-Silva

**Affiliations:** 1 Institute of Molecular Pathology and Immunology of the University of Porto (IPATIMUP), Porto, Portugal; 2 Eppley Institute for Research in Cancer and Allied Disease, Omaha, Nebraska, United States of America; 3 Medical Faculty, University of Porto, Porto, Portugal; University of Nebraska Medical Center, United States of America

## Abstract

**Background:**

Gastric carcinoma is the second leading cause of cancer-associated death worldwide. The high mortality associated with this disease is in part due to limited knowledge about gastric carcinogenesis and a lack of available therapeutic and prevention strategies. MUC1 is a high molecular weight transmembrane mucin protein expressed at the apical surface of most glandular epithelial cells and a major component of the mucus layer above gastric mucosa. Overexpression of MUC1 is found in approximately 95% of human adenocarcinomas, where it is associated with oncogenic activity. The role of MUC1 in gastric cancer progression remains to be clarified.

**Methodology:**

We downregulated MUC1 expression in a gastric carcinoma cell line by RNA interference and studied the effects on cellular proliferation (MTT assay), apoptosis (TUNEL assay), migration (migration assay), invasion (invasion assay) and aggregation (aggregation assay). Global gene expression was evaluated by microarray analysis to identify alterations that are regulated by MUC1 expression. *In vivo* assays were also performed in mice, in order to study the tumorigenicity of cells with and without MUC1 downregulation in MKN45 gastric carcinoma cell line.

**Results:**

Downregulation of MUC1 expression increased proliferation and apoptosis as compared to controls, whereas cell-cell aggregation was decreased. No significant differences were found in terms of migration and invasion between the downregulated clones and the controls. Expression of TCN1, KLK6, ADAM29, LGAL4, TSPAN8 and SHPS-1 was found to be significantly different between MUC1 downregulated clones and the control cells. *In vivo* assays have shown that mice injected with MUC1 downregulated cells develop smaller tumours when compared to mice injected with the control cells.

**Conclusions:**

These results indicate that MUC1 downregulation alters the phenotype and tumorigenicity of MKN45 gastric carcinoma cells and also the expression of several molecules that can be involved in tumorigenic events. Therefore, MUC1 should be further studied to better clarify its potential as a novel therapeutic target for gastric cancer.

## Introduction

Gastric cancer is one of the most common and life-threatening cancers worldwide (for review see [1]).The poor prognosis of this disease reflects our poor understanding of its etiological factors and pathogenesis and the lack of effective treatments.

MUC1 is a high molecular weight transmembrane protein that is expressed at the apical surface of most glandular epithelial cells [2]. MUC1 is overexpressed in almost 95% of cancer cells [3], a molecular pathological feature that is associated with carcinogenesis and poor prognosis [4], [5], [6], [7], [8]. Moreover, aberrant glycosylation and loss of apical expression of MUC1 have been reported for gastric carcinomas [9], [10], [11].

MUC1 protein consists of a highly variable extracellular domain composed of a variable number of tandem repeats (VNTR), and a highly conserved cytoplasmic domain (CD), which are both essential for MUC1-driven oncogenic activities [12], [13]. The MUC1 extracellular domain can be extensively glycosylated [14] and was shown to interact with several extracellular ligands, including ICAM-1[15] and galectin-3[16]. These interactions influence cell adhesion [16], motility and migration [17], [18], metastasis [19] and cell-cell aggregation [20], which contribute to the maintenance of a normal cell phenotype, and upon disregulation contribute to tumor progression. The MUC1 cytoplasmic domain (MUC1-CD) engages in signal transduction through several residues that can be phosphorylated by receptor tyrosine kinases (and other kinases), which in turn regulate MUC1-CD affinity to other mediators of signal transduction and transcriptional regulation [for review see [21] and [22]. MUC1-CD associates with molecules such as β-catenin, c-Src, Grb2/Sos, p53, GSK-3β, EGFR and PKC-δ [for review see [22], Lyn [23] , Lck and Zap 70 [24] , ER-α [25], NFKβ [26], c-Abl [27], ATM [28] and CAML [29], that regulate processes of cell survival, proliferation, apoptosis, adhesion, migration and cell-cell aggregation. These functions of MUC1 are known to contribute to tumor progression and poor survival of cancer patients [for review see [6], [30]. Nonetheless, the relevance of MUC1 in gastric cancer progression has not been previously investigated.

In the report presented here we used retrovirus-mediated transfection of short-hairpin RNAs (shRNA) to induce a stable downregulation of MUC1 in the gastric carcinoma- derived cell line MKN45. The effects of MUC1 downregulation were studied *in vitro* with respect to cell proliferation, apoptosis, migration, invasion and cell-cell aggregation. MUC1 downregulated cells were more proliferative and apoptotic than the controls and exhibited lower degrees of cell-cell aggregation. No significant differences were found in terms of cell migration and invasion. Global gene expression analysis, evaluated by oligonucleotide microarrays, identified several genes influenced by MUC1 downregulation that may contribute to the observed phenotypic alterations. *In vivo* studies have shown that MUC1 downregulation impacts tumor development.

## Materials and Methods

### Cell culture

A human cell line derived from diffuse-type gastric carcinoma – MKN45 (poorly differentiated adenocarcinoma, Japan Health Sciences Foundation [31]) was grown in RPMI 1640 containing Glutamax^TM^I and 25mM HEPES, supplemented with 10% fetal bovine serum (FBS) and 50 µg/ml gentamicin (Invitrogen). The packaging cell line PhoenixGP [32] was maintained in Dulbecco's Modified Eagle Medium containing Glutamax^TM^I, 4,500 mg/l D-Glucose and Sodium Pyruvate, supplemented with 10% FBS and 1% (v/v) penicillin/streptomycin. Stable MUC1 downregulated clones derived from MKN45 cells were grown in standard growth medium supplemented with 5 µg/ml puromycin (Sigma). After evaluation of MUC1 levels at different time points in culture, all the assays were performed considering the time of cell culture in which the downregulation was higher, at 96 hours of cell culture. Cells were grown at 37°C with 5% CO_2_ in humidified atmosphere.

### MUC1 downregulation strategy

MUC1 downregulated cells were produced using a retroviral expression system with short hairpin RNAs. Briefly, a 21-nucleotide sequence of the MUC1 gene, with no homology to other DNA sequences detected in a BLAST search, was chosen according to standard RNAi rules [33]. The scramble control was designed and tested for homology in a BLAST search as well. Sense and antisense oligos (Proligo) were ligated and inserted in the pSUPER.retro.puro vector (Oligoengine). The oligos used were the following: MUC1 Exon 2 (*sense*: GATCCCCACCTCCAGTTTAATTCCTCTTCAAGAGAGAGGAATT AAACTGGAGGTTTTTTA; *antisense*: AGCTTAAAAAACCTCCAGTTTAATTCCTCTC TCTTGAAGAGGAATTAAACTGGAGGTGGG; the MUC1-cDNA target region is underlined) and scramble control (*sense*: GATCCCCATCACCTTCGTACTCCTTA TTCAAGAGATAAGGAGTACGAAGGTGATTTTTTA, *antisense*: AGCTTAAAAAATC ACCTTCGTACTCCTTATCTCTTGAATAAGGAGTACGAAGGTGATGGG; the “unpaired”-cDNA target region is underlined). The MUC1 specific target or the scrambled control constructs were transfected into PhoenixGP packaging cell line by calcium-phosphate mediated transfection and transfected cells were selected using puromycin. Stable transfectants were seeded in a 6-well plate (1x10^6^cells/well) and incubated for 24 hours at 32°C. The media containing the virus was collected, filtered through a 0.45 µm filter to remove remnant cells, and used to infect MKN45 cells, during 24 hours at 37°C. The viral supernatant was then replaced by the standard growth medium and cells were incubated 48 hours at 37°C. Efficiently transduced cells were selected and grown in standard media supplemented with puromycin. Two independent MUC1 downregulated clones (C1 and C2) were isolated and expanded for three times using cloning rings. 

### Immunofluorescence

MKN45 cells at 96h in culture were harvested, seeded in 12-well slides (Cell Line) and air-dried overnight at room temperature. Cells were then fixed in ice cold acetone for 5 minutes, washed twice with PBS and blocked with normal rabbit serum (DAKO) diluted 1∶5 in 10% bovine serum albumin (BSA) for 30 minutes. Serum was then replaced by the MUC1 monoclonal antibody HMFG1 (NovoCastra) diluted 1∶50 in 5% BSA, and incubated overnight at 4°C. After three washes with PBS, cells were incubated with a rabbit anti-mouse FITC labeled antibody (DAKO) diluted 1∶70 in 5% BSA for 30 minutes in the dark at room-temperature. Cells were washed 3 times with PBS and mounted in vectashield (Vectorlabs). Images were acquired in a Leica DMIRE2 fluorescent microscope. Results are representative of three independent experiments.

### Protein extraction and Western blot

MKN45 cells were cultured in 60-mm dishes to 80–90%-confluence at 96h in culture. After washing twice with PBS, lysis buffer (10mM Tris pH 7.4, 150mM NaCl, 0.1% (p/v) SDS, 1mM PMSF, 1% (v/v) Triton X-100) was added and cells were scraped. Lysates were incubated on ice for 1 hour and centrifuged for 2 minutes at 4°C to collect the supernatants. Protein content was assessed by the bicinchoninic acid method (Pierce), as described in the manufacturer's instruction manual. Protein extracts were analyzed by a 4–10% SDS-PAGE (Invitrogen), transferred to a nitrocellulose membrane (Amersham Biosciences), and blotted overnight at 4°C with anti MUC1-Ab5 monoclonal antibody (1∶300, ThermoScientific), anti-beta-actin polyclonal antibody (1∶8,000, Sigma), anti ERK1/2(1∶1,000, Cell Signaling Technology) and anti β-catenin (1∶1,000, BD Transduction Laboratories) in 5% non-fat milk in TBS-0.1%Tween20 (Sigma). Membranes were washed 3 times with TBS-0.1%Tween20 and the primary antibodies were revealed using goat anti-mouse peroxidase-conjugated antibody (1∶1,000, DAKO) in 5% non-fat milk in TBS-0.1%Tween20, followed by ECL detection kit (BioRad). Results are representative of three independent experiments.

### RNA extraction and Real-Time PCR

Total RNA was isolated from MKN45 cells at 96h in culture using TriReagent^TM^ (Sigma), according to the manufacturer's instructions. 5 µg of RNA were primed with random hexamers (Invitrogen) and reverse transcribed with Superscript II (Invitrogen) in a final volume of 20 µl. 2 µl of a 1∶10 dilution of cDNA were amplified with 300nM of each primer and SYBRGreen (Applyed BioSystems) in a final volume of 20 µl, using the fluorescence reader ABI Prism 7000. Each sample was run in duplicate. The primers used were the following: MUC1 (s*ense*: CTCCTTTCTTCCTGCTGCTG, *anti*s*ense*: CTGGAGAGTACGCTGCTGGT) and 18S (*sense*: CGCCGCTAGAGGTGAAATTC, *anti*s*ense*: CATTCTTGGCAAATGCTTTCG), and their specificity was confirmed using the software BLASTn on-line and by melt curve analysis. For each sample, the level of 18S RNA was measured and used for normalization of target genes abundance. Relative mRNA levels were then calculated using the comparative C_t_ method [34]. Data is expressed as a ratio of the results obtained with each clone and the scramble control, from three independent experiments. Statistical analysis was performed using the Mann-Whitney test.

### MTT proliferation assay

Cells were plated in triplicate in 96-well plates at 5,000 cells per well and incubated at normal conditions for 96h for MKN45 cells. At each time point, the medium was removed and cells incubated with 20 µl of MTT solution (5mg/ml, Sigma) for 3 hours at normal conditions. MTT was removed and 200 µl of DMSO were added to each well to dissolve formazan. Finally, formazan optical density was measured using a microplate reader at a wavelength of 540nm. The relative growth was defined as the following formula: Relative Growth  =  (A_540nm_ at Tn / A_540nm_ at T0_24h_). Data is expressed as a ratio of the results obtained with each clone and the scramble control, from three independent experiments. Statistical analysis was performed using the Mann-Whitney test.

### Terminal Transferase dUTP Nick End Labeling (TUNEL) assay

Post-confluent cells at 96h in culture were harvested and fixed with 4% paraformaldehyde in PBS for 15 minutes. Fixed cells were seeded in 12-well slides (Cell Line) and air-dried overnight at room temperature. Following washing with PBS, cells were permeabilized with ice-cold freshly-made PBSTrCit solution (PBS + 0.1%TritonX + 0.1% Sodium Citrate) for 2 minutes on ice. Cells were washed again twice, and incubated with TUNEL reaction mix (enzyme solution, label solution and dilution buffer, 1∶9∶10, In Situ Death Detection Kit, Fluorescein, Roche) for 1 hour at 37°C. Two additional washing steps were performed and slides were mounted in Vectashield with DAPI (Vectorlabs). Results were analyzed under a Leica DMIRE2 fluorescent microscope and data is expressed as a ratio of the results obtained with each clone and the scramble control, from three independent experiments. Statistical analysis was performed using the Mann-Whitney test.

### Migration assay

MKN45 cells were cultured in 60-mm dishes for a full-confluence at 96h in culture. The epithelial cells monolayer was then washed with PBS and wounded with a 10 µl micropipette tip. Non-adherent cells were removed by washing twice with PBS. Images of cells at the edge of the wound were acquired automatically at 20x magnification in a Leica DMIRE2 fluorescence microscope with a Leica DFC Twain camera for 144 frames at 10-minute intervals (corresponding to 24 hours) controlled by Leica FW4000 software. Frames from 0, 6, 12, 18 and 24 hours were used to quantify the percentage of migration: a grid of 50x30 squares was used to fulfill the wound space and the percentage of migration was calculated by the number of squares occupied by cells at each time point. Data is expressed as a ratio of the results obtained with each clone and the scramble control, from three independent experiments. Statistical analysis was performed using the Mann-Whitney test.

### Matrigel invasion assay

Cell invasion was studied by using BD Biocoat™ Matrigel™ invasion chambers with 8-µm size pores (BD Biosciences), according to the manufacturer's instructions. MKN45 cells at 96h in culture were harvested and seeded in duplicate at 250,000 cells per insert (sized for 24-well plates) in 1% FBS containing medium, and 20% FBS containing medium was added to the bottom of the growth well, as an attractant. Cells were allowed to invade for 22 hours (37°C, 5% CO_2_ atmosphere). The non-invading cells were then swabbed from the top of the inserts and the invading cells on the lower surface were fixed with 100% methanol and stained with DAPI for 15 minutes in the dark. The membranes were removed and cells were counted under a Leica DMIRE2 fluorescence microscope. Data are expressed as a ratio of the results obtained with each clone and the scramble control, from three independent experiments. Statistical analysis was performed using the Mann-Whitney test.

### Cell-cell Aggregation assay

MKN45 cells at 96h in culture were harvested and seeded in duplicate at 250,000 cells per well in 24-well plates. Plates were placed at 37°C with constant stirring (150rpm) for 1 and 2 hours. Cells were fixed with 100 µl of 25% glutaraldehyde at time zero and at the end of the incubation. Aggregates were photographed under a light microscope and isolated cells were counted (cells in duplicates were counted as isolated cells). The aggregation index was defined as the following formula: Aggregation index  = 1- (number of isolated cells at Tn / number of isolated cells at T0). Data are expressed as a ratio of the results obtained with each clone and the scramble control, from three independent experiments. Statistical analysis was performed using the Mann-Whitney test.

### Gene expression analysis

The expression of 12,135 genes in MUC1 downregulated clones and the respective scramble control were evaluated following the same protocol as before [35]. Briefly, following RNA extraction (as described previously), cDNA was obtained by reverse transcription, during which labeled nucleotides were incorporated: MKN45-C1 and MKN45-C2 cDNAs were labeled with Cy3 (green emission) and MKN45-SC control with Cy5 (red emission). After hybridization, the mixture was hybridized with the array overnight and then the array was digitalized with the ScanArray4000 (Perkin-Elmer) system and fluorescence analyzed by the QuantArray software package (Perkin-Elmer). 

Normalization and background subtraction were performed and ratios for downregulated clones /Scrambled Control and Scrambled Control/downregulated clones were calculated using Microsoft Excel software. Gene expression with a ratio higher than 2 was considered statistically significant.

All data is MIAME compliant and that the raw from the microarray experiments were uploaded onto the Gene Expression Omnibus Database http://www.ncbi.nlm.nih.gov/geo (Geo accession numbers: GSM717858 and GSM717859).

### *In Vivo* tumor growth Assays

Six-week-old female N:NIH(s)II:nu/nu nude mice were obtained previously from the Medical School, University of Cape Town in 1991 and maintained and housed at IPATIMUP Animal House at the Medical Faculty of the University of Porto, in a pathogen-free environment under controlled conditions of light and humidity. Males and females, aged 6–8 weeks, were used for *in vivo* experiments. Animal experiments were carried out in accordance with the Guidelines for the Care and Use of Laboratory Animals, directive 86/609/EEC. Mice were subcutaneously injected in the dorsal flanks using a 25-gauge needle with 1x10^5^ of MKN45-SC (2 male and 2 female mice) or MKN45-C2 cells (3 male and 3 female mice). Mice were weighed, and tumor width and length were measured with calipers every week. Mice were euthanized 21 days after cell injection, at the time when the first tumor reached maximum allowable volume. For statistical analysis, the Mann Whitney test-StatView Software version 5.0 (SAS Institute, Cary, NC) was used. A *P* value of less than 0.05 was considered as statistically significant.

## Results

### MUC1 downregulation by shRNA

**Figure 1:**
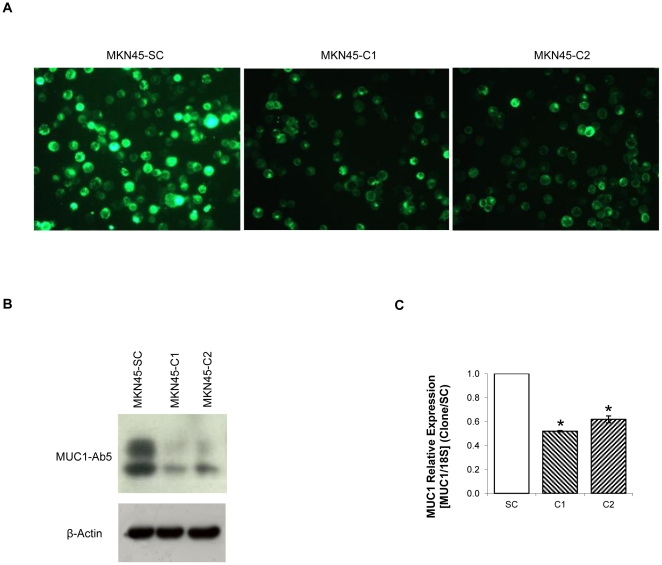
MUC1 downregulation by shRNA. (**A**) MUC1 detection by immunofluorescence with HMFG-1 antibody in MKN45-C1 and MKN45-C2 and MKN45-SC control; (**B**) MUC1 protein detection by western-blot with MUC1-Ab5 antibody of total protein extracts from MKN45-C1 and MKN45-C2 and MKN45-SC control; (**C**) Quantification of MUC1 RNA in MKN45-C1 and MKN45-C2 and MKN45-SC control by real-time PCR. MUC1 expression was corrected to the house-keeping gene 18S and normalized to the data obtained with the scrambled control. *P<0.01.

We established two independent MUC1 downregulated clones, MKN45-C1 and MKN45-C2 and one scramble control, MKN45-SC. MUC1 downregulation was verified by immunofluorescence (Figure 1A), Western Blot (Figure 1B) and Real-Time PCR (Figure 1C).

There was a significant downregulation of MUC1 expression in MKN45-C1 and MKN45-C2 clones as compared to the MKN45-SC control. The expression of MUC1 at the protein level was detected with two different antibodies, one that binds the VNTR extracellular domain (HMFG-1, Figure 1A) and other that recognizes a 14–28 KDa sequence in MUC1 cytoplasmic domain (MUC1-Ab5, Figure 1B). Both showed a significant reduction in the amount of MUC1 protein in MKN45-C1 and MKN45-C2 clones when compared to the scramble control. Real-Time PCR results indicate that the MUC1 downregulation was 48% (MKN45-C1) and 38% (MKN45-C2) (Figure 1C). MUC1 RNA levels were evaluated at 48, 72 and 96h of cell culture and the highest downregulation occurred at 96h (results not shown).

### Effects of MUC1 downregulation on MKN45 cells

#### Cell proliferation

**Figure 2:**
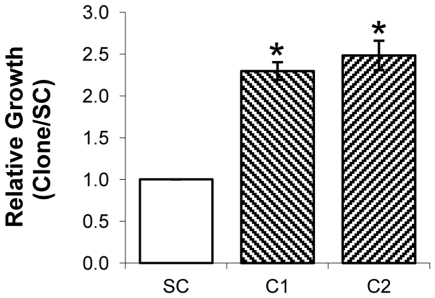
Quantification of cell proliferation by MTT assay. Quantification of metabolically active cells by MTT assay in MKN45-C1 and MKN45-C2 clones and MKN45-SC control at 96h in culture. Data from 24 hours was used to set time zero and results were normalized to the data obtained with the scrambled control.*P<0.01.

MKN45-C1 and MKN45-C2 cells showed significantly increased proliferation rates (*P*<0.01) when compared to the MKN45-SC control (2.29 and 2.48 *vs* 1), when evaluated by MTT assay (Figure 2).

#### Cell apoptosis

**Figure 3:**
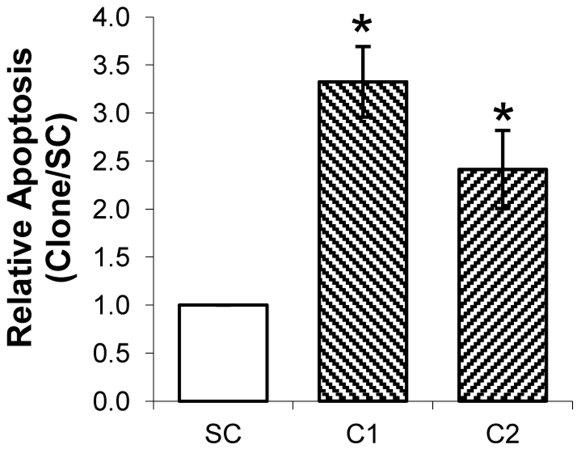
Quantification of apoptotic cells by TUNEL assay. Apoptosis of MKN45-C1 and MKN45-C2 and scramble control (SC) were evaluated at 96h in culture by the TUNEL assay. Results were normalized to the data obtained with the scrambled control. *P<0.01.

MKN45-C1 and MKN45-C2 cells showed significantly increased levels of apoptosis (*P*<0.01) when compared to the MKN45-SC control (3.32 and 2.41 *vs* 1), when evaluated by a TUNEL assay (Figure 3).

#### Cell-cell aggregation

**Figure 4:**
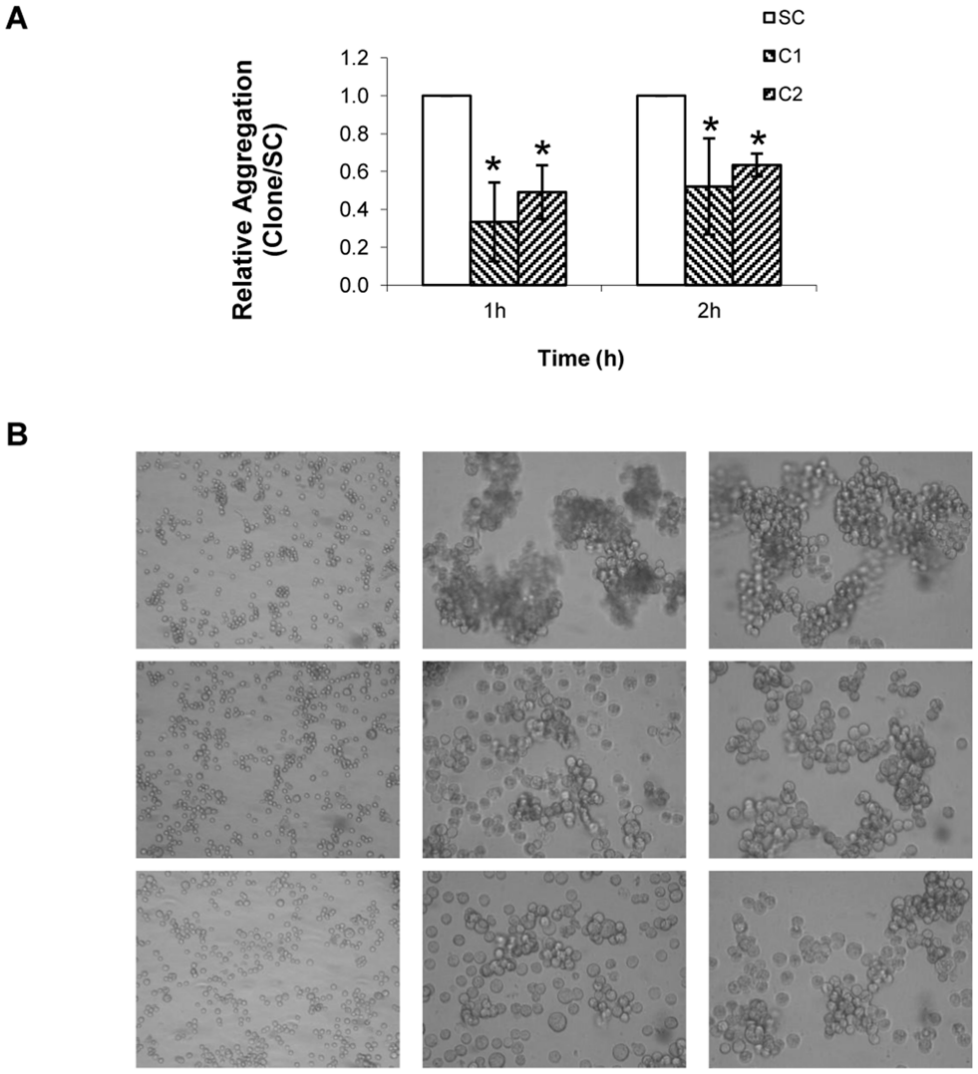
Quantification of cell-cell aggregation. (**A**)Quantification of the cell-cell aggregation index in MKN45-C1 and MKN45-C2 and MKN45-SC control. The cell-cell aggregation index was assessed by the observed decrease in the number of isolated cells over time, and normalized to the data obtained with the scrambled control. *P<0.01; (**B**) Images of the aggregates formed after 1 and 2 hours of constant stirring. First column shows isolated cells at time 0h (20x magnification) and second and third columns show aggregates formed after 1h and 2h of incubation (40x magnification), in MKN45-C1 and MKN45-C2 and MKN45-SC control.

MKN45-C1 and MKN45-C2 cells showed significantly decreased cell-cell aggregation levels (*P*<0.01), when compared to the MKN45-SC control (0.34 and 0.49 *vs* 1 at 1h; 0.52 and 0.64 *vs* 1 at 2h), when evaluated by a cell aggregation assay (Figures 4A and 4B).

#### Cell migration and invasion

There were no significant differences in cell migration and invasion of MKN45-C1 and MKN45-C2 cells when compared to the MKN45-SC control, when evaluated by motility and invasion assays (results not shown).

### Effects of MUC1 downregulation in MKN45 cells gene expression

The observed phenotypic modifications associated were likely due in part to alterations in signal transduction pathways mediated by MUC1-CD, since overexpression of MUC1 has been shown to modulate gene expression through reprograming transcription of multiple genes [36], [37]. We evaluated the net effects of downregulating MUC1 in the MKN45 gastric carcinoma cell line by performing a global analysis of gene expression by oligonucleotide microarrays (Table 1). The results revealed that a number of genes that influence proliferation, migration, invasion and motility were differentially expressed in MKN45-C1 and MKN45-C2 and the MKN45-SC control. The most significant differences were found for TCN1, KLK6 and ADAM29 (>10 fold upregulated between the MKN45-C1 and MKN45-C2 clones and the MKN45-SC control) and LGALS4, TSPAN8 and SHPS-1 (>3.5 fold downregulated between the MKN45-C1 and MKN45-C2 clones and the MKN45-SC control).

**Table 1:** Oligonucleotide microarray results by comparison between MKN45-C1/MKN45-C2 and MKN45-SC control cells, by order of magnitude.

**Genes upregulated >2 fold in MUC1 downregulated clones**
Transcobalamin 1 (TCN1)
Kallikrein-related peptidase 6 (KLK6)
Desintegrin and metalloproteinase 29 (ADAM29)
Keratoepithelin (TGFBI)
MRP family of ATP transport member 2 (ABCC2)
Amyloid beta precursor-like protein 2 (APLP2)
Mitochondrial ATP synthase (ATP5I)
Sulfide dehydrogenase like protein (SQRDL)
Sarcoglycan, epsilon (SGCE)
Hypothetical protein (FLJ20323)
Galectin 1 (LGALS1)
Proline-histidine rich protein (PHLDA1)
Trypsin 2 (PRSS2)
Mesotrypsin (PRSS3)
SP2 transcription factor (SP2)
Ubiquitin-conjugating enzyme (UBE2L6)
Vitellogenic-like carboxypeptidase (CPVL)
**Genes downregulated >2 fold in MUC1 downregulated clones**
Galectin 4 (LGALS4)
Tetraspanin 8 (TSPAN8)
Tyrosine phosphatase SHP substrate (SHPS-1)
Polymerase (DNA-directed), delta 4 (POLD4)
H2B histone family, member J (HIST1H2BH)
H2B histone family, member T (HIST1H2Bk)
Carcinoembryonic antigen-related cell adhesion molecule 5 (CEACAM5)
Annexin IV (ANXA4)
Intercellular adhesion molecule 4 (ICAM4)
DEAD (Asp-Glu-Ala-Asp) box polypeptide 39 (DDX39)
Apolypoprotein B (APOBEC2)
Clusterin (CLU)
GDP-mannose 4,6-dehydratase (GMDS)
Serine/threonine kinase 38 like (STK38L)
CD55 (CD55)
Apolipoprotein B-catalytic polypeptide-like 3C (APOBEC3C)
Cell adhesion related-molecule (CDON)
Villin-1 (VIL1)

MKN45-C1 and MKN45-C2 and the MKN45-SC control were analysed by oligonucleotide microarrays. Listed are genes with expression increased or decreased more than 2 fold in both MUC1 downregulated clones when compared to the control.

### *In vivo* tumor growth assays

**Figure 5:**
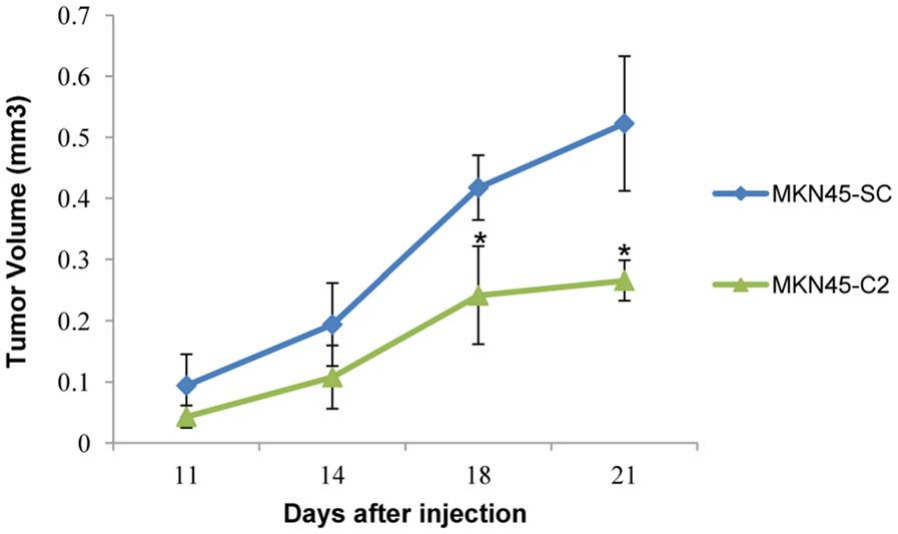
Study of the tumorigenicity of MKN45 gastric carcinoma cells *in vivo*. Tumor growth curves. 1×10^5^ cells were subcutaneously injected in mice at day 0. The curves show tumor growth until day 21, the day on which all mice were sacrificed.* P<0.05, when compared to the MKN45-SC control cell line.

*In vivo* tumorigenicity assays showed that mice injected with MUC1-downregulated cells (MKN45-C2) developed smaller and slower-growing tumors, when compared to mice injected with the MKN45-SC control cells (Figure 5).

## Discussion

In the work presented here, we evaluated the effects of MUC1 downregulation on cancer-related properties of MKN45 gastric carcinoma cells. Stable downregulation of MUC1 expression was achieved in MKN45 gastric carcinoma cell line by RNA interference. MUC1 contributes to tumor progression of adenocarcinomas and therefore its downregulation was predicted to affect the malignant properties of cancer cells, including proliferation, apoptosis, migration, invasion and cell-cell aggregation.

We found that proliferation was significantly increased in MUC1 downregulated clones MKN45-C1 and MKN45-C2 when compared to the control MKN45-SC. Similar studies with breast and pancreatic carcinoma cell lines have shown similar [38] and opposite [35], [38], [39] results. In different tumor models, MUC1 was shown to regulate cell proliferation by interacting with several proteins such as ER-α, β-catenin and EGFR [25], [40], [41]. However, for gastric carcinoma cells, such interactions have not been investigated. Results obtained by an oligonucleotide microarray analysis showed that expression of molecules affecting cell proliferation such as KLK6 and LGAL-4 [42], [43], [44] were significantly altered in MKN45-C1 and MKN45-C2 clones when compared to the MKN45-SC control. KLK6 expression was increased in MKN45-C1 and MKN45-C2 clones, whereas LGAL4 expression was decreased and these differences may explain the observed differences in proliferation. The mechanisms by which KLK6 and LGAL4 expression is altered in MKN45-C1 and MKN45-C2 when compared to MKN45-SC control remains to be elucidated.

Another important observation was that apoptosis was significantly increased in MKN45-C1 and MKN45-C2 clones when compared to the MKN45-SC control. MUC1 was previously shown to mediate a pro-apoptotic response in hamster ovary cells [45] and it was also attributed with anti-apoptotic functions in myeloma, breast and colorectal carcinoma cell lines [46], [47], [48]. However, little is known about the influence of MUC1 on cell apoptosis in gastric carcinoma cells.

No significant differences were found between MUC1-C1 and C2 clones and MKN45-SC control with respect to cell migration and invasion. This is in contrast to previous findings in which MUC1 was shown to influence cell migration in breast, cervical, renal and pancreatic carcinoma cell lines [18], [49], [50], [51] and cell invasion in breast, lung, gastrointestinal, hepatic and pancreatic carcinoma cell lines [3], [52], [53].

Cell-cell aggregation was decreased in MKN45-C1 and MKN45-C2 clones when compared to the MKN45-SC control. Previous studies have shown that overexpression of different forms of MUC1 can lead to an increase or a decrease in cell-cell aggregation in a pancreatic carcinoma cell line [54], whereas others have shown that MUC1 downregulation induces an increase of cell-cell aggregation in an oral carcinoma cell line [41]. MUC1 interactions with other adhesion molecules have been shown to contribute to both adhesive [55], [56] and anti-adhesive [57], [58], [59] properties of cells. Our results showed that MUC1 plays a relevant role in MKN45 cell-cell aggregation, contributing to gastric cells adhesive properties.

Another possibility is that signaling through the MUC1-CD influences gene expression, which in turn affects the phenotypic properties of the MKN45 cell line. By oligonucleotide microarray analysis we found alterations in the transcriptional profile of cells following MUC1 downregulation when compared to control cells. These alterations are likely due to MUC1 downregulation, since MUC1 has been shown to directly conduct signals that alter the transcriptional program of tumor cells [36], [37], [60]. MUC1 cytoplasmic domain can be phosphorylated in several sites, modulating its interaction with cell signalling partners and transcription factors [21]. The phosphorylation of MUC1-CD will be dependent on the amount and availability of its signaling partners and therefore on the cell type in question. We found significant alterations in the expression levels of several genes, mainly TCN1, KLK6, ADAM29, LGALS4, TSPAN8 and SHPS-1. Some of these molecules have functions not yet fully clarified yet others are known to be associated with cell proliferation and migration, including KLK6, LGAL4 and SHPS-1 [42], [43], [59], [61], [62], invasion, including KLK6 [59] and motility, including LGAL4 [43]. MUC1 may be facilitating the transcription of these genes and therefore be contributing to the observed phenotypic alterations observed.

*In vivo* assays confirmed that cells with decreased levels of MUC1 form smaller and slower-growing tumors than the control cells. This result emphasizes that MUC1 contributes to gastric tumor progression in the context of the multicellular environment of tumor growth in vivo.

MUC1 overexpression has been associated with the neoplastic progression of several tumors, including the acquisition of invasive and metastatic properties. Phenotypic studies in cell models other than gastric cancer have suggested that MUC1 influences events such as proliferation, apoptosis, migration, invasion, adhesion and cell-cell aggregation. Previous studies of MUC1 in breast carcinogenesis models show mixed results for different breast cancer cell lines [38], which reinforces the relevance of the molecular context on the MUC1-mediated cancer progression. The effects of MUC1 in gastric carcinogenesis will thus be dependent on MUC1 and the molecules interacting with MUC1, which will significantly differ between cell lines. Evaluation of different gastric cell lines will complement the data regarding the impact of MUC1 gastric carcinogenesis.

The work presented here shows for the first time that MUC1 expression influences proliferation, apoptosis and cell-cell aggregation of MKN45 gastric carcinoma cells. The results are consistent with the view that MUC1 modulates different signaling pathways in a manner that is dependent on the expression and activity of other regulatory mechanisms and molecules, which are influenced by the cellular and biological context of the cell type that is overexpressing MUC1.
